# Patient empowerment and general self-efficacy in patients with coronary heart disease: a cross-sectional study

**DOI:** 10.1186/s12875-018-0749-y

**Published:** 2018-05-30

**Authors:** Anita Kärner Köhler, Pia Tingström, Tiny Jaarsma, Staffan Nilsson

**Affiliations:** 1 Department of Social and Welfare Studies (ISV/OMV), Linköping University, Norrköping, Sweden; 2 Department of Medical and Health Sciences (IMH), Linköping University, Linköping, Sweden; 3 Primary Health Care, Region Östergötland, Linköping, Sweden

**Keywords:** Patient empowerment, General self-efficacy, Coronary heart disease, Self-rated health, Well-being, Primary health care

## Abstract

**Background:**

In managing a life with coronary heart disease and the possibility of planning and following a rehabilitation plan, patients’ empowerment and self-efficacy are considered important. However, currently there is limited data on levels of empowerment among patients with coronary heart disease, and demographic and clinical characteristics associated with patient empowerment are not known.

The purpose of this study was to assess the level of patient empowerment and general self-efficacy in patients six to 12 months after the cardiac event. We also aimed to explore the relationship between patient empowerment, general self-efficacy and other related factors such as quality of life and demographic variables.

**Methods:**

A sample of 157 cardiac patients (78% male; age 68 ± 8.5 years) was recruited from a Swedish hospital. Patient empowerment was assessed using the SWE-CES-10. Additional data was collected on general self-efficacy and well-being (EQ5D and Ladder of Life). Demographic and clinical variables were collected from medical records and interviews.

**Results:**

The mean levels of patient empowerment and general self-efficacy on a 0–4 scale were 3.69 (±0.54) and 3.13 (±0.52) respectively, and the relationship between patient empowerment and general self-efficacy was weak (*r* = 0.38). In a simple linear regression, patient empowerment and general self-efficacy were significantly correlated with marital status, current self-rated health and future well-being. Multiple linear regressions on patient empowerment (Model 1) and general self-efficacy (Model 2) showed an independent significant association between patient empowerment and current self-rated health. General self-efficacy was not independently associated with any of the variables.

**Conclusions:**

Patients with a diagnosis of coronary heart disease reported high levels of empowerment and general self-efficacy at six to 12 months after the event. Clinical and demographic variables were not independently associated with empowerment or low general self-efficacy. Patient empowerment and general self-efficacy were not mutually interchangeable, and therefore both need to be measured when planning for secondary prevention in primary health care.

**Trial registration:**

NCT01462799.

**Electronic supplementary material:**

The online version of this article (10.1186/s12875-018-0749-y) contains supplementary material, which is available to authorized users.

## Background

Coronary heart disease is the major cause of premature death among men and women in Europe [1]. Yearly, around 30,000 Swedes suffer a myocardial infarction [2] and among patients who survive, about 20% suffer a secondary cardiovascular event in the first year. Around 50% of major coronary events occur among those with a previous diagnosis of myocardial infarction [3]. Regular exercise, smoking cessation and the use of cardiovascular preventive drugs are effective preventive actions that reduce mortality and re-infarction rates [4]. Yet, risk factors remain significant in patients, even after initiation of secondary prevention. A study of patients with coronary heart disease showed that 48.6% continued to smoke 1.35 years in median time after the cardiovascular event, almost two out of three patients were physically inactive, 38% were obese, more than 40% had hypertension and 80.5% had hypercholesterolemia [5].

Cardiac rehabilitation and secondary prevention, which are confirmed to be effective and safe in the management of clinically stable patients with coronary heart disease, are two other kinds of support accessible during the recovery process. Therefore, it is problematic that only 20–50% of the eligible patients [6] attend cardiac rehabilitation programmes that could facilitate smoking cessation and taking up physical exercise, which are really effective preventive actions [7] that reduce mortality after myocardial infarction [8, 9].

For patients recovering from a myocardial infarction, restenosis after percutaneous coronary intervention, or coronary artery bypass surgery, achieving control of their own health has been viewed as a struggle, including negotiating the management of daily problems [10]. Bodily experiences make cardiac patients uncertain and frightened of recurrence and death. Patients’ questions about how long the effects of the cardiac surgery will last also demonstrate their uncertainty. It seems that after revascularisation, patients are not always aware that they are chronically ill, and this may have consequences for the management and practice of self-care [11]. Additionally, patients experience, for example, constraining somatic and social incentives, affecting their capacity to perform physical activity and to follow programmes of drug treatment [12]. If patients felt safe and in control based on their understanding of their heart disease during cardiac rehabilitation and were inclined to manage self-care with support, their recovery could proceed more smoothly. Patients’ recovery after coronary heart disease seems complex and challenging, and we need to know more about key factors that facilitate this recovery.

Patient empowerment (PE) is viewed as a key factor for improving health outcomes, enhancing communication between patients and health professionals, bringing about better adherence to treatment regimes, and ensuring the efficient use of primary health resources. This is outlined as a specific intention by the World Health Organization’s (WHO) Regional Office for Europe in Health 2020 [13]. According to this policy framework, which provides main strategies and priorities to support European action for health and well-being, people are increasingly seen as co-producers of their own health, and need to be empowered to take control of the determinants of their own health [13]. The WHO defines PE as a process where patients understand their role, are given the knowledge and skills by the health care provider to perform a task in an environment where there is an awareness of community and cultural differences, and where patients are encouraged to participate [14]. A recent concept analysis of PE stated that:

*“Individual patient empowerment is a process that enables patients to exert more influence over their individual health by increasing their capacities to gain more control over issues they themselves define as important* [15] (p. 1927).”

Moreover, Castro et al. [15] found that PE is situated on several levels (micro-, meso- and macro-) and could be approached by the patient, the health care provider or the health care system.

Self-efficacy (SE), a concept derived from social cognitive theory, concerns people’s beliefs in their capacity to exercise control over events that affect their lives [16]. SE beliefs influence how people think, feel, motivate themselves and act [17]. It has been viewed as relevant in relation to the fact that people and populations who believe they will succeed are more likely to attempt a new behaviour. To achieve SE, a person might perform a task that was previously successful within their capabilities (mastery experience), watch someone with whom they can identify performing a task successfully (social modelling), receive positive feedback/verbal persuasion relating to the task from someone or interpret physiological or affective states, with some or all of these factors being present before SE is accomplished [18]. SE is a well-studied concept within cardiac rehabilitation. Relationships between perceived SE [19] and personal goals during cardiac rehabilitation have been positively associated with physical exercise six and 12 months after discharge [20], with food choices after a tailored one-year intervention at an outpatient clinic [21], with smoking habits two to four weeks after discharge [22], and with health-related quality of life two years post-MI [23]. General SE (GSE) has received attention in research, and it is defined as the belief in one’s competence to cope with a broad range of stressful and challenging demands. GSE appears to be a universal construct yielding meaningful relations with other psychological constructs and having a positive impact on health behaviours [24]. Patients with high GSE recovered better one week after cardiac surgery and experienced a higher quality of life six months later compared with their low-GSE counterparts [25]. Thus, it is important to consider GSE when predicting behavioural intentions and health behaviour change after an event of coronary heart disease.

We argue that GSE and PE are important to consider at an individual level, for managing life with coronary heart disease, as well as at an organisational level, for arranging secondary cardiac rehabilitation in primary health care. However, PE is not well-studied in cardiac rehabilitation.

We therefore assessed the level of GSE and PE in patients who had been diagnosed with coronary heart disease six to 12 months ago. Based on previous literature and theoretical foundation we also explored the relation between other related factors such as gender, age marital status, education, well-being, self-rated health, cardiovascular risk factors and diseases, in relation to PE and GSE.

## Methods

### Setting

In this cross-sectional study, we recruited patients with coronary heart disease in south-east Sweden between September 2011 and November 2014. A detailed description of the study procedures is given in a design article [26], but a summary is provided below.

### Recruitment

The patients were invited to the COR-PRIM study, an ongoing five-year prospective single- centre randomised controlled study (NCT01462799), performed in a primary health care setting in south-eastern Sweden. The current analysis uses the baseline data of the patients. The recruitment was conducted by the Vrinnevi Hospital heart unit in Norrköping, Sweden, from the electronic medical record, based on the following criteria: Inclusion criteria were: (i) patients with coronary heart disease verified by myocardial infarction and/or percutaneous coronary intervention and/or coronary artery bypass surgery six to 12 months before the planned start of the interventions, (ii) patients who were stable regarding their cardiac condition and taking optimised cardiac medication that had not substantially changed during the previous month, (iii) patients who had completed heart school in hospital, and were listed at one of six specific primary health care centres. Exclusion criteria were: planned coronary artery bypass surgery or other conditions demanding continuing cardiologic care; for example: ongoing contact with heart failure clinic due to drug titration, life expectancy ≤ one year, documented psychiatric disease causing difficulties in cooperating with other people, obvious abuse of alcohol or narcotics. Patients were also excluded if they were unable to read or communicate in the Swedish language.

**Fig. 1:**
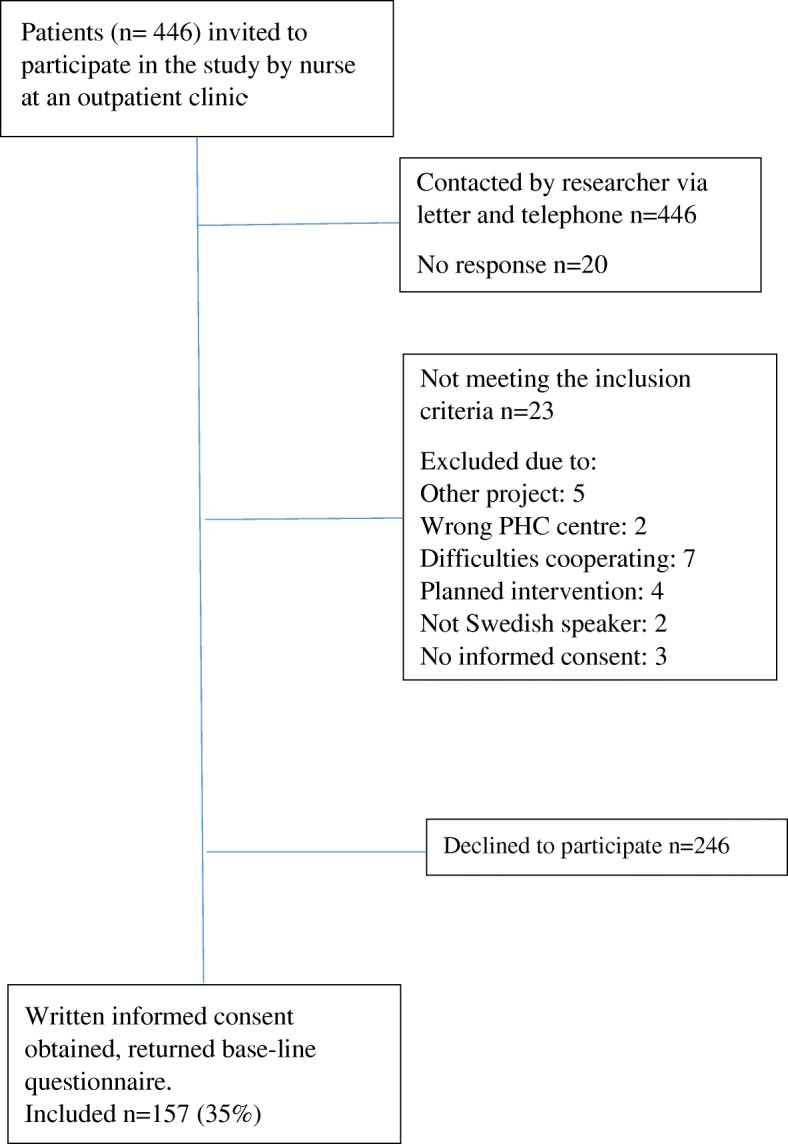
Sampling frame

The patients were informed about the study during a visit to a nurse. The nurse asked the patients if the researcher in charge of the project could contact them by letter and telephone call in order to further inform them and discuss possible recruitment with them. Of 446 patients invited, 157 consented to participate (response rate 35%), completed and sent back questionnaires at baseline before randomisation and before any intervention was started (Fig. 1).

### Patient questionnaires

Patient empowerment was assessed by the SWE-CES-10 questionnaire, which was developed to survey PE in patients with coronary heart disease. This questionnaire was originally based on the SWE-DES-23, which is a valid and reliable tool for assessing PE in diabetes mellitus (DM) and rheumatic disease (RA) [27–29]. The SWE-DES-23 was shortened to become the SWE-DES-SF-10 and was found to be reliable in relation to the original version. The author of the scale allowed us to replace the word ‘diabetes’ with ‘heart disease’ in all 10 items and agreed that further psychometric testing was not needed before using the scale in patients with cardiac disease. The SWE-CES-10 has four subscales assessing different components of patient empowerment: 1) Goal achievement and overcoming barriers to goal achievement, 2) Self-awareness, 3) Managing stress, 4) Assessing dissatisfaction and readiness to change. The items are scored on a five-point Likert scale ranging from ‘strongly disagree’ (1) to ‘strongly agree’ (5). A higher value indicates a stronger empowerment [30].

Self-efficacy was assessed by the General Self-Efficacy Scale (GSES, Additional file 1) [31], on which items are scored on a four-point Likert scale ranging from ‘not at all true’ (1) to ‘exactly true’ (4). A higher score indicates a higher GSE, which predicts the ability to cope with daily problems and the ability to adapt after experiencing various stressful life events [32]. High reliability, stability and construct validity of the GSES scale are confirmed in earlier studies [33, 34].

Well-being was assessed by the Cantril Ladder, a single-item indicator of well-being. This scale is presented as a ladder with steps numbered from zero at the bottom to 10 at the top, where 10 represents the best possible life for the participant, and zero the worst. The participants are asked on which step of the ladder they feel they stood one year ago, on which step they stand at present, and on which step they will stand one year in the future. The Ladder of Life has been used in large population studies, and tested for reliability, concurrent and predictive validity [35]. It has also been used in elderly patients recovering from an acute coronary event [36] and in a randomised study of patients with coronary heart disease [37].

Self-rated health was measured by the Visual Analogue Scale within the EQ5D (Additional file 2). It produces scores of 0–100, with higher scores indicating a better overall quality of life [38].The EQ5D is considered to be a reliable and valid instrument for use in patients with stable coronary heart disease [39].

### Demographic and clinical data

Patients’ baseline data on: type of cardiovascular event, days of treatment of hypertension and hyperlipidaemia, smoking, presence of comorbidities (DM, hypertension, chronic obstructive pulmonary disease, stroke, heart failure) were collected from the Swedeheart® register and the medical records about two weeks after discharge from the hospital. Age, sex, education, residential area, job position and marital status were collected from patient questionnaires (Table 1). The patients were also asked to self-report, in free text their diseases. Cardiovascular diseases and DM were excluded. Self-reported comorbidity was categorised by the researchers as: comorbidities of all kinds or comorbidity affecting mobility. This was done in order to catch the total experience of burden of disease.

**Table 1:** Baseline characteristics of patients suffering from a coronary heart disease event 6–12 months ago. Comparison of self-reported high or low patient empowerment (SWE-CES-10^1^) and general self-efficacy (GSES^a^)

	Total *n* = 157 n (%), mean (SD)	SWE-CES-10 ≤ 3.6 *n* = 77 n (%), mean (SD)	SWE-CES-10 > 3.6 *n* = 72 n (%), mean (SD)	*p*-value	GSES ≤ 2.95 *n* = 48 n (%), mean (SD)	GSES > 2.95 *n* = 98 n (%), mean (SD)	*p*-value
Gender, M/F	122/35	58/19	58/14	0.442	36/12	76/22	0.732
Age, years	68.7 (8.5)	68.4 (8.6)	68.7 (8.4)	0.828	68.7	68.8	0.919
Residential area							
City	74 (47.1)	39 (50.6)	30 (41.7)		24 (50.0)	43 (43.9)	
Rural or small town	83 (52.9)	38 (49.4)	42 (48.3)	0.272	24 (50.0)	55 (56.1)	0.486
Education							
Compulsory education^b^	84 (53.5)	40 (51.9)	39 (54.2)		25 (52.1)	54 (55.1)	
Upper secondary school	31 (19.7)	14 (18.2)	16 (22.2)		10 (20.8)	20 (20.4)	
University	38 (24.2)	21 (27.3)	17 (23.6)	0.493	13 (27.1)	23 (23.5)	0.869
Job position							
Employed	26 (16.9)	10 (13.0)	16 (22.2)		9 (18.8)	15 (15.3)	
Self-employed	15 (9.7)	10 (13.0)	5 (6.9)		4 (8.3)	10 (10.2)	
Disabled pensioner	9 (5.8)	6 (7.8)	3 (4.2)		5 (10.4)	4 (4.1)	
Retired pensioner	104 (67.5)	50 (64.9)	48 (66.7)	0.295	30 (62.5)	68 (69.4)	0.531
Marital status							
Cohabitating	115 (73.2)	53 (68.8)	59 (81.9)		33 (68.8)	75 (76.5)	
Living alone	40 (25.5)	24 (31.2)	13 (18.1)	0.064	15 (31.3)	23 (23.5)	0.314
Time from cardiac event to start of study group, days	284 (74)	274 (75)	291 (70)	0.147	277 (67)	289 (78)	0.362
Smoking, current	19 (12.1)	10 (13.0)	8 (11.1)	0.726	7 (14.6)	10 (10.2)	0.438
Cardiac event one year before study inclusion^c^							
Myocardial infarction	86 (54.8)	43 (55.8)	41 (56.9)		25 (52.1)	56 (57.1)	
Other	71 (45.2)	34 (44.2)	31 (43.1)	0.892	23 (47.9)	42 (42.9)	0.563
Cardiac event one year before study inclusion							
PCI only	110 (70.1)	55 (71.4)	50 (69.4)		34 (70.8)	68 (69.4)	
CABG, CABG+PCI or MI without revascularisation	47(29.9)	22 (28.6)	22 (30.6)	0.791	14 (29.2)	30 (30.6)	0.858
Number of previous myocardial infarctions							
1	15 (10.1)	9 (11.7)	6 (8.3)		4 (8.3)	8 (8.2)	
2	3 (2.0)	1 (1.3)	2 (2.8)		2 (4.2)	1 (1.0)	
3	2 (1.3)	1 (1.3)	1 (1.4)	0.841	0 (0)	2 (2.0)	0.468
Angina pectoris, diagnosed	47 (29.9)	26 (33.8)	19 (26.4)	0.327	17 (35.4)	28 (28.6)	0.400
CCS^d^							
I	98 (62.4)	53 (75.7)	41 (64.1)		26(61.9)	66 (73.3)	
II	27 (17.2)	9 (12.9)	16 (25.0)		9 (21.4)	17 (18.9)	
III	11 (7.0)	6 (8.6)	5 (7.8)		5 (11.9)	5 (5.6)	
IV	4 (2.5)	2 (2.9)	2 (3.1)	0.345	2 (4.8)	2 (2.2)	0.430
Diabetes Mellitus	25 (15.9)	9 (11.7)	14 (19.4)	0.190	11 (22.9)	13 (13.3)	0.139
Hypertension	75 (47.8)	37 (48.1)	36 (50.0)	0.812	26 (54.2)	47 (48.0)	0.481
Medication for hypertension before study start, days	1376 (649–2094)^e^	1542 (673–2107)^e^	1270 (645–1883)^e^	0.383	1459 (649–2149)^e^	1187 (649–1912)^e^	0.641
COPD^f^	15 (9.6)	9 (11.7)	6 (8.3)	0.496	5 (10.3)	9 (9.2)	0.812
Hyperlipidaemia	56 (35.7)	31 (40.3)	24 (33.3)	0.381	23 (47.9)	29 (29.6)	0.030
Days with treatment for hyperlipidaemia before study start	1038 (420–1884)^e^	695 (338–2156)^e^	1150(507–1799)^e^	0.513	1061 (341–1740)^e^	1067 (456–2038)^e^	0.324
Comorbidity, all kinds	60 (38.2)	26 (33.8)	31 (43.1)	0.244	16 (33.3)	40 (40.8)	0.382
Comorbidity, affecting mobility	17 (10.8)	10 (13.0)	7 (9.7)	0.531	6 (12.5)	9 (9.2)	0.535

^a^Dichotomized

^b^Less than 10 years in school

^c^Current, basis for study inclusion

^d^Canadian Cardiovascular Society scale for grading angina pectoris

^e^Median (IQR)

^f^Chronic obstructive pulmonary disease

### Statistical analyses

Descriptive statistics were used to characterise the study population. Means, medians and standard deviations are presented for patients’ baseline data involving clinical and demographical measures. Chi-square tests were used to compare dichotomised variables. The SWE-CES-10 (Patient Empowerment Scale) was dichotomised based on the median value in the present sample (md = 3.6). For GSES (Additional file 1), the mean value for population data [40] was used as a cut-off value (m = 2.95) when dichotomised. Simple linear and multiple regression models were used where the two main outcome variables, GSES and PE, were considered dependent. In order not to overlook effects, the SWE-CES-10 and GSES variables were used as continuous variables in multiple linear regression analysis (Model 1 and 2). Simple linear regressions tested the association of PE and GSES with sex, age, marital status, education, well-being (Cantril’s Ladder) and self-rated health (EQ5D). The significant variables from the simple linear regressions, together with sex and age, were used in Models 1 and 2. A *p*-value < 0.05 was considered significant.

## Results

**Fig. 2:**
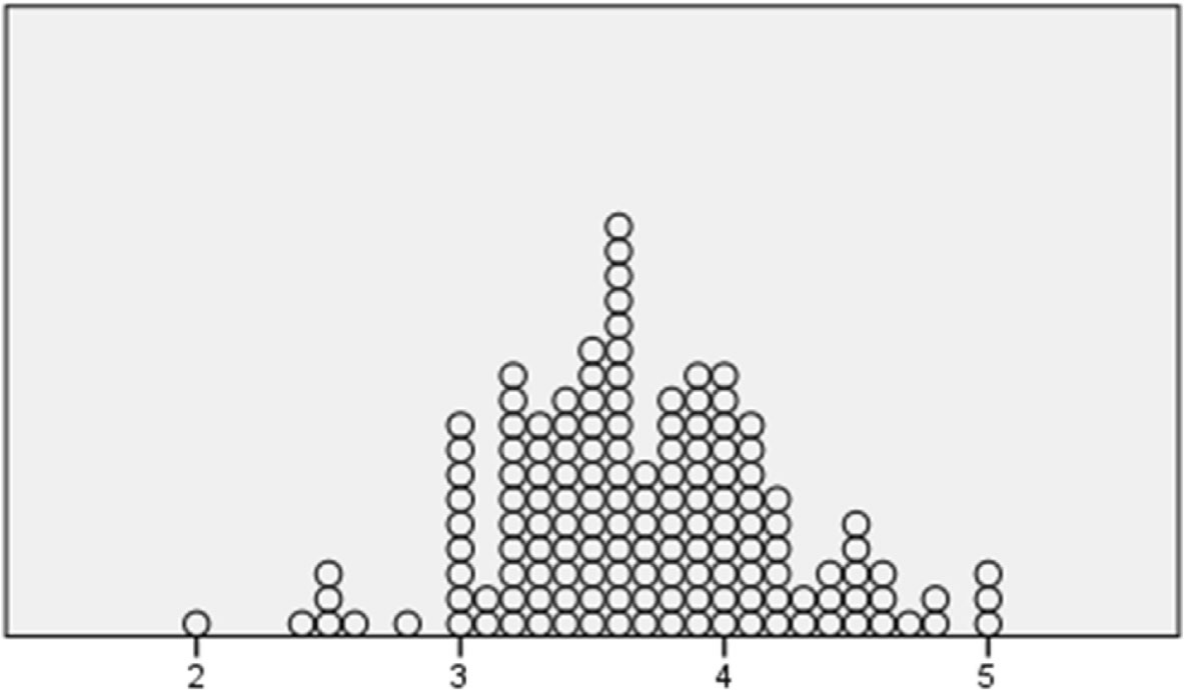
Distribution of empowerment SWE-CES. Scale 1–5 (*n* = 150)

**Fig. 3:**
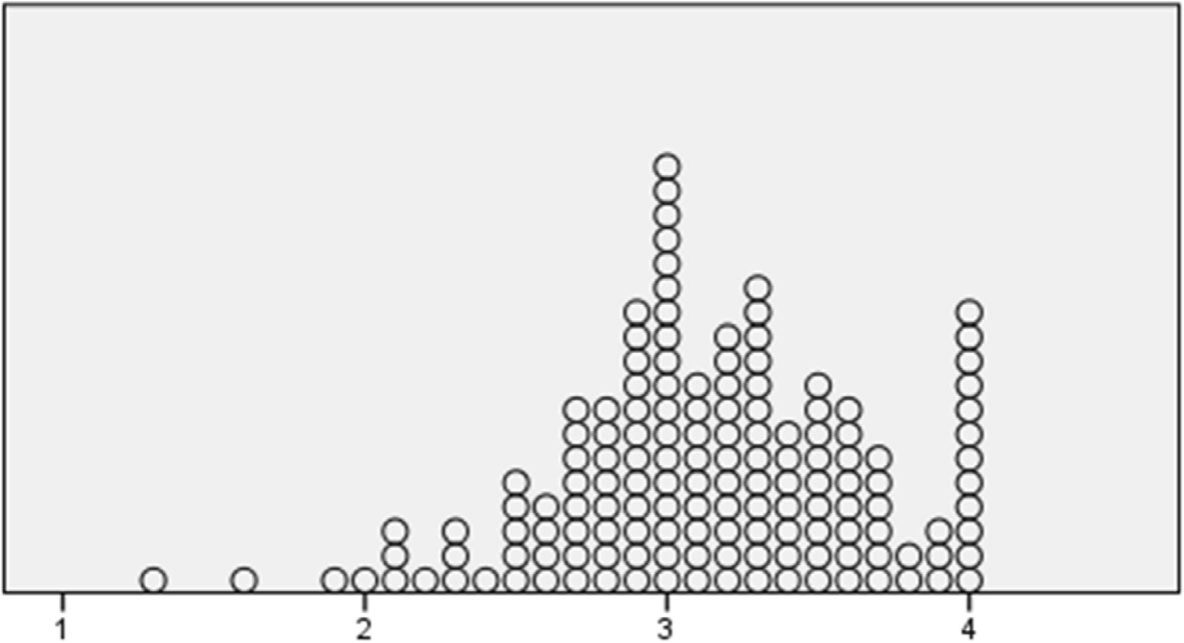
Distribution of self-efficacy. Scale 1–4 (*n* = 144)

Of the individuals included, three quarters were men, and the mean age for the whole group was 68.7 (±8.5) (range 47–87) years (Table 1). There were equal proportions living in rural or small town areas or in the city. Half of the group (54.8%) had suffered a myocardial infarction, and 70.1% of all participants had been treated with percutaneous coronary intervention. Twenty participants had had a previous myocardial infarction. In total, 29.9% of the participants had been diagnosed with angina pectoris, with 2.5% having severe angina (CCS IV). The mean value for the participants’ (*n* = 149) self-rated total PE was 3.69 (±0.54) (theoretical range 1 low and 5 high). Mean values for subscales were: Goal achievement 3.76 (±0.65), Self-awareness 3.90 (±0.99), Managing stress 3.47 (±0.86), Readiness to change 3.57 (±0.76). The mean value for GSES was 3.13 (±0.52) (*n* = 146). Distributions are shown in Figs. 2 and 3.

The correlation between the SWE-CES and GSES was, *r* = 0.381 (*p* < 0.000). When SWE-CES and GSES were dichotomised, there were no significant differences between participants rating high or low on these variables, with the exception of hyperlipidaemia and GSE (Table 1).

### Patient empowerment, general self-efficacy and related variables

Gender, age, marital status, education, self-rated health and well-being were considered to be possibly related to PE. All of these variables were first tested with simple linear regression. The variables that were significantly related to PE, together with gender and age, were used in the multiple linear regression model. Consequently, the variable ‘education’ was excluded from Model 1. The same procedures as applied in PE were used when analysing GSES. There were significant correlations between PE and marital status, self-rated health (EQ5D) and well-being (Ladder of Life) in a simple linear regression. In the multiple linear regression, Model 1, the association between PE and self-rated health (EQ5D) remained significant (Table 2). For GSE, there were significant univariate correlations for all variables except age and education. We excluded education in the multiple linear model. In the multiple linear regression, Model 2, none of the variables were significantly associated with GSE (Table 3).

**Table 2:** Simple and multiple correlation for patient empowerment (SWE-CES-10^a^). R^2^ 0.125, adjusted R^2^ 0.091

	Simple linear regression				Multiple linear regression			
Independent variables	Unstandardized Beta coefficient	95% CI		*p*-value	Unstandardized Beta coefficient	95% CI		*p*-value
Gender, M^2^/F	−0.174	− 0.382	0.034	0.100	−0.92	− 0.302	0.119	0.392
Age, years	0.004	−0.007	0.014	0.474	0.008	−0.002	0.018	0.114
Education	−0.016	−0.115	0.082	0.743	NA	NA	NA	NA
Cohabitating^b^/ Living alone	0.201	−0.400	−0.003	0.047	−0.088	− 0.295	0.120	0.405
Current self-rated health (scale 0–100)^c^	0.010	0.005	0.014	< 0.001	0.007	0.002	0.013	0.014
Well-being (scale 0–10)^d^	0.060	0.014	0.106	0.011	0.021	−0.035	0.076	0.463

^a^Continuous variable, ^b^Reference, ^c^ EQ5D, ^d^ Ladder of life, *NA* Not applicable

**Table 3:** Simple and multiple correlation for dependent variable general self-efficacy (GSES^a^). R^2^ 0.111, adjusted R^2^ 0.076

	Simple linear regression				Multiple linear regression			
Independent variables	Unstandardised Beta coefficient	95% CI		*p*-value	Unstandardised Beta coefficient	95% CI		*p*-value
Gender, M^2^/F	−0.202	−0.0399	− 0.005	0.045	− 0.145	−0.350	0.061	0.166
Age, years	0.000	−0.010	0.010	0.960	0.005	−0.006	0.015	0.371
Education	0.024	−0.074	0.123	0.628	NA	NA	NA	NA
Cohabitating^b^/ Living alone	−0.246	−0.434	− 0.057	0.011	− 0.111	−0.317	0.095	0.288
Current self-rated health (scale 0–100)^c^	0.008	0.004	0.013	< 0.001	0.004	−0.002	0.010	0.178
Well-being (scale 0–10)^d^	0.065	0.021	0.109	0.004	0.040	−0.015	0.095	0.149

^a^Continuous variable, ^b^Reference, ^c^ EQ5D, ^d^ Ladder of life, *NA* Not applicable

## Discussion

This is the first study assessing PE and GSE and related variables in a cardiac population. The levels of GSE and PE were high, and GSE in this group was similar when compared to a general population [40, 41] as well as for patients with other chronic illnesses [34]. This might indicate that levels of PE or GSE are not related to chronic conditions.

The main message of this study is that we were not able to find demographic and clinical variables that could be used as predictors of low PE or GSE. We cannot predict how patients with coronary heart disease rate PE or GSE based on variables such as age, sex, marital status, or type of cardiovascular event. We found a significant association between self-rated health and PE. The higher the patients rated their health, the higher the PE. However, self-rated health was not related to GSE. Our findings do not support the hypothesis of Bravo et al. (2015), who suggested that patients scoring high for PE would have better reported outcomes, e.g. be better adapted to their condition; have better quality of life; report higher levels of well-being and satisfaction with life and achieve some independence in relation to their health care [42].

The mean level of PE in total did not differ from other studies on chronic diseases, such as rheumatoid arthritis (RA) [43] and DM [28]. On some of the subscales of PE, the cardiac population scored higher compared to RA on self-awareness (3.90) vs (3.5) and lower compared to RA/DM on readiness to change (3.57) vs. (3.7); higher compared to DM on goal- achievement (3.76) vs (3.64) [28, 43]. An interpretation of this finding could be that the cardiac population know enough for making self-care choices, and when and where to get support if needed. They are goal- oriented and can over-come barriers to reach the goals. However, part of the self-care causes dissatisfaction and to some extent they are cautiously awaiting self-care change. This might be due to lack of motivation despite them being knowledgeable.

According to the literature review [18], few studies have investigated the connection between SE and PE. We found a weak correlation between PE and GSE, indicating that these cannot replace each other. GSE was not related to educational level, civil status or well-being, either. It is vital for people to acquire SE in order to be able to handle adversity and the struggles they encounter in life [17]. We therefore argue that it is important to identify patients with low GSE and PE, as this might place them at risk of failing to reach goals in their cardiac rehabilitation plan. By measuring PE and GSE, we can identify the patients that are in need of support during cardiac rehabilitation. As addressed by WHO, PE is a key factor for improving health outcomes, enhancing communication between patients and health professionals, and adherence to treatment regimens [13].

There is a paradigm shift taking place, as clinical encounters have traditionally focussed on persuading patients to comply with taking medication as prescribed [44], and PE is aiming instead at a collaboration between health care professionals and patients, assisting the latter in becoming self-aware of the considerations, needs and barriers to changing their lifestyle, managing their illness, and using resources to solve problems in their daily lives [45]. There is convincing evidence that empowerment-based self-management interventions can improve PE and also health outcomes. For example, PE was significantly stronger among people with RA diseases after problem-based learning programmes compared to a control group six months post-intervention [43]. A meta-analysis of empowerment-based interventions showed improvements in HbA1C, SE and empowerment levels in patients with chronic metabolic diseases [46]. Such interventions can be designed to facilitate patients’ self-management, and PE is also considered as a valuable measurable patient-reported outcome for depression, DM and asthma [47, 48] and, among survivors from cancer, using information technology to improve PE [49]. However, further randomised trials are needed to evaluate the effects of interventions aimed at improving GSE and PE to change/manage self-care. Longitudinal trials could help us to define at what scores additional support is warranted [26].

### Strengths and limitations

The present study has some limitations. Firstly, we used cross-sectional data that was collected during an intervention study. However, despite this shortcoming, this is to our knowledge the first study that has investigated PE in patients with coronary heart disease.

Secondly, we were unable to include more than half of the invited patients in the study. Some gave reasons such as feeling too old, living abroad for half of the year, difficulties in travelling to the group meetings, or social circumstances, but we did not structurally collect data on the non-participants, as in accordance with ethical legislation, reasons for non-participation could not be collected. We cannot therefore say whether the participants differ significantly from the non-participants. In total, 35% of eligible patients were enrolled and that may be considered as a small number, and as highly selected, as they from the start were positive and wanted to take part in the COR-PRIM-study [26], which is quite a demanding programme, with group discussions in primary care over a period of one year and a long follow-up time of five years. Internal drop-outs were low, and in comparison with Jelinek et al. [6] who enrolled 20–50% of eligible patients to cardiac rehabilitation, the number of participants in our study is not low.

## Conclusion

PE and GSE are not mutually interchangeable, and therefore both need to be measured when planning for secondary prevention in primary health care. The results showed that we cannot predict a high-risk group based on demographic variables, e.g. patients’ sex, age, marital status or education. Therefore, in order to support PE and GSE, all patients need to be offered follow-up, and we are inclined to say that it should be personalised, with a focus on the patients’ beliefs, needs and goals.

## Additional files

Additional file 1: Questionnaire measuring General Self-efficacy (PDF 89 kb)

Additional file 2: Questionnaire measuring Self-rated health (PDF 110 kb)

## Abbreviations

COR-PRIM: Coronary heart disease-primary care
CR: Cardiac rehabilitation
EQ5D: European Quality of Life–Five Dimensions
GSE (S): General Self-Efficacy (Scale)
PE: Patient Empowerment
SE: Self-efficacy
SWE-CES: Swedish Cardiac Empowerment Scale

## References

[1] Hartley A, Marshall D, Salciccioli J, Sikkel M, Maruthappu M, Shalhoub J (2016). Trends in mortality from ischemic heart disease and cerebrovascular disease in Europe: 1980-2009. Circulation.

[2] Socialstyrelsen: Statistik om hjärtinfarkter 2016. In: Hälso- och sjukvård; 2017. Retrieved 2018-05-17 from http://www.socialstyrelsen.se/publikationer2017/2017-10-23.

[3] Jernberg T, Hasvold P, Henriksson M, Hjelm H, Thuresson M, Janzon M (2015). Cardiovascular risk in post-myocardial infarction patients: nationwide real world data demonstrate the importance of a long-term perspective. Eur Heart J.

[4] Perk J, De Backer G, Gohlke H, Graham I, Reiner Z, Vershuren WMM, Albus C, Benlian P, Boysen G, Cifkova R (2012). European guidelines on cardiovascular disease prevention in clinicl practice (version 2012). Eur Heart J.

[5] Kotseva K, Wood D, De Bacquer D, De Backer G, Rydén L, Jennings C, Gyberg V, Amouyel P, Bruthans J, Castro Conde A (2015). EUROASPIRE IV: a European Society of Cardiology survey on the lifestyle, risk factor and therapeutic management of coronary patients from 24 European countries. Eur J Prev Cardiol.

[6] Jelinek M, Thomson D, Ski C, Bunker S, Vale M (2015). 40 years of cardiac rehabilitation and secondary prevention in post-cardiac ischaemic patients. Are we still in the wilderness?. Int J Cardiol.

[7] Anderson LJ, Taylor RS (2014). Cardiac rehabilitation for people with heart disease: an overview of Cochrane systematic reviews. Int. J Cardiol.

[8] Critchley J, Capewell S (2003). Smoking cessation for the secondary prevention of coronary heart disease. Cochrane Database Syst Rev.

[9] Wilson K, Gibson N, Willan A, Cook D (2000). Effect of smoking cessation on mortality after myocardial infarction: meta-analysis of cohort studies. Arch Intern Med.

[10] Kristofferzon M-L, Löfmark R, Carlsson M (2007). Striving for balance in daily life: experiences of Swedish women and men shortly after a myocardial infarction. J Clin Nurs.

[11] Odell A, Grip L, Hallberg R-M (2006). Restenosis after percutaneous coronary intervention (PCI): experiences from the patients' perspective. Eur J Cardiovasc Nurs.

[12] Kärner A, Tingstrom P, Abrandt Dahlgren M, Bergdahl B (2005). Incentives for lifestyle changes in patients with coronary heart disesae. J Adv Nurs.

[13] WHO/Europe: Health 2020: the European policy for health and well-being - About Health 2020: Retrieved 2018-05-17 from http://www.euro.who.int/en/health-topics/health-policy/health-2020-the-european-policy-for-health-and-well-being/about-health-2020.

[14] WHO: Patient empowerment and health care. In*.*; 2009: Retrieved 2017–12-07 from http://www.ncbi.nlm.nih.gov/books/NBK144022/

[15] Castro EM, Van Regenmortel T, Vanhaecht K, Sermeus W, Van Hecke A (2016). Patient empowerment, patient participation and patient-centeredness in hospital care: a concept analysis based on a literature review. Patient Educ Couns.

[16] Bandura A (1995). Self-efficacy in changing societies.

[17] Zulkosky K (2009). Self-efficacy: a concept analysis. Nurs Forum.

[18] Rawlett K (2014). Journey from self-efficacy to empowerment. Health Care.

[19] Schwarzer R, Fuchs R, Bandura A (1995). Changing risk behaviors and adopting health behaviors: the role of self-efficacy beliefs. Self-efficacy in changing societies.

[20] Slovinec D'Angelo M, Pelletier L, Reid R, Huta V (2014). The roles of self-efficacy and motivation in the prediction of short- and long term adherence to exercise among patients with coronary heart disease. Health Psychol.

[21] Sol B, van der Graaf Y, van Petersen R, Visseren F (2011). The effects of self-efficacy on cardiovascular lifestyle. Eur J Cardiovasc Nurs.

[22] Bakker E, Nijkamp M, Sloot C, Berndt N, Bolman C (2015). Intention to abstain from smoking among cardiac rehabilitation patients - The role of attitudes, self-efficacy and craving. J Cardiovasc Nurs.

[23] Brink E, Alsén P, Herliz J, Kjellgren K, Cliffordson C (2012). General self-efficacy and health-related quality of life after myocardial infarction. Psychol, Healt & Med.

[24] Bandura A, Bandura A (1995). Exercise of personal and collective efficacy in changing societies. Self-efficacy in changing societies.

[25] Schröder KEE, Schwarzer R, Konertz W (1998). Coping as a mediator in recovery from cardiac surgery. Psychol Health.

[26] Kärner A, Nilsson S, J T, Andersson A, Wiréhn A-B, Wodlin P, Hjelmfors L, Tingstrom P (2012). The effect of problem-based learning in patient education after an event of CORONARY heart disease - a randomised study in PRIMARY health care: design and methodology of the COR-PRIM study. BMC Fam Pract.

[27] Arvidsson S, Bergman S, Arvidsson B, Fridlund B, Tingström P (2012). Psychometric properties of the Swedish rheumatic disease empowerment scale, SWE-RES-23. Musculoskeletal Care.

[28] Leksell J, Funnel M, Sandberg G, Smide B, Wiklund G, Wikblad K (2007). Psychometric properties of the Swedish diabetes empowerment scale. Scand J Caring Sci.

[29] Barr PJ, Scholl I, Bravo P, Faber MJ, Elwyn G, McAllister M: Assessment of patient empowerment - a systematic review of measures. PLoS One. 2015;10(5):e0126553. 10.1371/journal.pone.0126553. 10.1371/journal.pone.0126553 PMC4430483 25970618

[30] Anderson R, Fitzgerald J, Gruppen L, Funnel MM, Oh M: the diabetes empowerment scale- short-form (DES_SF). Diabetes Care 2013, 26(5):1641–1642. 10.2337/diacare.26.5.1641-a 12716841

[31] Koskinen- Hagman M, Schwarzer R, Jerusalem M: Swedish version of the general self-efficacy scale. In*.*, vol. 2017: http://userpage.fu-berlin.de/health/swedish.htm; 1999.

[32] Schwarzer R, Jerusalem M, Weinman J, Wright S, Johnston M (1995). Generalized self-efficacy scale. Measures in health psychology: A user's portfolio Causal and control beliefs.

[33] Leganger A, Kraft P, R⊘ysamb E: perceived self-efficacy in health behaviour research: conceptualisation, measurement and correlates. Psychol Health 2000, 15(1):51–69.

[34] Luszczynska A, Scholz U, Schwarzer R (2005). The general Sel-efficacy scale: multicultural validation studies. Aust J Psychol.

[35] Andrews F, Withey S (1976). Social indicators of well-being: American's perceptions of life quality.

[36] Ståhle A, Mattsson E, Rydén L, Unden AL, Nordlander R (1999). Improved physical fitness and quality of life following training of elderly patients after acute coronary events. A 1 year follow-up randomized controlled study. Eur Heart J.

[37] Tingström P, Kamwendo K, Bergdahl B (2005). Effects of a problem-based learning rehabilitation programme on quality of life in patients with coronary artery disease. Eur J Cardiovasc Nurs.

[38] The EuroQol G: EuroQol* - a new facility for the measurement of health-related quality of life. Health Policy 1990, 16:199–208. 10.1016/0168-8510(90)90421-9 10109801

[39] De Smedt D, Clays E, Doyle F, Kotseva K, Prugger C, Pająk A, Jennings C, Wood D, De Bacquer D (2013). Validity and reliability of three commonly used quality of life measures in a large European population of coronary heart disease patients. Int J Cardiol.

[40] Scholz U, Gutierrez Dona B, Sud S, Schwarzer R (2002). Is general self-efficacy a universal construct? Psychometric findings from 25 countries. Eur J Psychol Ass.

[41] Swarzer, R. Renner, B. Health-specific self-efficacy scales**.** 2014. https://www.researchgate.net/publication/251801350_Health-Specific_Self. Efficacy_Scales. Accessed 2017–11-10.

[42] Bravo P, Edwards A, Barr PJ, Scholl I, Elwyn G, McAllister M (2015). Conceptualising patient empowerment: a mixed methods study. BMC Health Serv Res.

[43] Arvidsson S, Bergman S, Arvidsson B, Fridlund B, Tingström P (2012). Effects of a self-care promotion problem-based learning programme in people with rheumatic diseases: a randomized controlled study. J Adv Nurs.

[44] Funnell MM (2016). Patient empowerment: what does it really mean?. Patient Educ Couns.

[45] Anderson RM, Funnel MM (2005). Patient empowerment: reflections on the challenge of fostering the adoption of a new paradigm. Patient Educ Couns.

[46] Kuo CC, Lin CC, Tsai FM (2014). Effectiveness of empowerment-based self-management interventions on patients with chronic metaboloc diseases: a systematic review and meta-analysis. Evid Based Nurs.

[47] McAllister M, Dunn G, Payne K, Davies K, Todd C (2012). Patient empowerment: the need to consider it as a measurable patient- reported outcome for chronic conditions. BMC Health Serv Res.

[48] Tsai A, Morton S, Mangione C, Keeler E (2005). A meta-analysis of interventions to improve care for chronic illnesses. Am J Manag Care.

[49] Groen W, Kuijpers W, Oldenburg H, Wouters M, Aaronson N, Harten W (2015). Empowerment of cancer survivors through information technology: an intergrative review. J Med Internet Res.

