# Supplementary material for: Patient empowerment and general self-efficacy in patients with coronary heart disease: a cross-sectional study
Source: BMC Fam Pract. 2018 May 30;19:76. doi: 10.1186/s12875-018-0749-y (PMC5975271; doi:10.1186/s12875-018-0749-y)
Supplement: Supplementary file 1 — Questionnaire measuring General Self-efficacy (PDF 89 kb) [file 12875_2018_749_MOESM1_ESM.pdf]

# The General Self-Efficacy Scale (GSE)

Authors: Ralf Schwarzer & Matthias Jerusalem

The Scale is available athttp://userpage.fu-berlin.de/~health/engscal.htm

| | |
|---|---|
| 1 | I can always manage to solve difficult problems if I try hard enough. |
| 2 | If someone opposes me, I can find the means and ways to get what I want. |
| 3 | It is easy for me to stick to my aims and accomplish my goals. |
| 4 | I am confident that I could deal efficiently with unexpected events. |
| 5 | Thanks to my resourcefulness, I know how to handle unforeseen situations. |
| 6 | I can solve most problems if I invest the necessary effort. |
| 7 | I can remain calm when facing difficulties because I can rely on my coping abilities. |
| 8 | When I am confronted with a problem, I can usually find several solutions. |
| 9 | If I am in trouble, I can usually think of a solution. |
| 10 | I can usually handle whatever comes my way. |

**Response Format:**

1 = Not at all true   2 = Hardly true   3 = Moderately true   4 = Exactly true
